# Subcutaneous ω-Conotoxins Alleviate Mechanical Pain in Rodent Models of Acute Peripheral Neuropathy

**DOI:** 10.3390/md19020106

**Published:** 2021-02-11

**Authors:** Md. Mahadhi Hasan, Hana Starobova, Alexander Mueller, Irina Vetter, Richard J. Lewis

**Affiliations:** 1 Division of Chemistry and Structural Biology, Institute for Molecular Bioscience, The University of Queensland, Brisbane, QLD 4072, Australia; mahadhi.hasan@imb.uq.edu.au (M.M.H.); h.starobova@imb.uq.edu.au (H.S.); a.mueller@imb.uq.edu.au (A.M.); i.vetter@uq.edu.au (I.V.); 2 School of Pharmacy, The University of Queensland, Brisbane, QLD 4102, Australia

**Keywords:** ω-conotoxins, chemotherapy-induced peripheral neuropathy, postsurgical pain, intraplantar administration

## Abstract

The peripheral effects of ω-conotoxins, selective blockers of N-type voltage-gated calcium channels (Ca_V_2.2), have not been characterised across different clinically relevant pain models. This study examines the effects of locally administered ω-conotoxin MVIIA, GVIA, and CVIF on mechanical and thermal paw withdrawal threshold (PWT) in postsurgical pain (PSP), cisplatin-induced neuropathy (CisIPN), and oxaliplatin-induced neuropathy (OIPN) rodent models. Intraplantar injection of 300, 100 and 30 nM MVIIA significantly (*p* < 0.0001, *p* < 0.0001, and *p* < 0.05, respectively) alleviated mechanical allodynia of mice in PSP model compared to vehicle control group. Similarly, intraplantar injection of 300, 100, and 30 nM MVIIA (*p* < 0.0001, *p* < 0.01, and *p* < 0.05, respectively), and 300 nM and 100 nM GVIA (*p* < 0.0001 and *p* < 0.05, respectively) significantly increased mechanical thresholds of mice in OIPN model. The ED_50_ of GVIA and MVIIA in OIPN was found to be 1.8 pmol/paw and 0.8 pmol/paw, respectively. However, none of the ω-conotoxins were effective in a mouse model of CisIPN. The intraplantar administration of 300 nM GVIA, MVIIA, and CVIF did not cause any locomotor side effects. The intraplantar administration of MVIIA can alleviate incision-induced mechanical allodynia, and GVIA and MVIIA effectively reduce OIPN associated mechanical pain, without locomotor side effects, in rodent models. In contrast, CVIF was inactive in these pain models, suggesting it is unable to block a subset of N-type voltage-gated calcium channels associated with nociceptors in the skin.

## 1. Introduction

ω-Conotoxins are 24–30 amino-acid-long venom peptides mostly found in fish hunting cone snails from the Conidae family [1,2]. These basic peptides have a net positive charge ranging between +5 and +7, with three disulfide bonds formed between conserved cysteine residues stabilizing their globular structure [3]. The unique feature of ω-conotoxins is that they selectively block voltage-gated calcium (Ca_V_) channels, including Ca_V_2.2 channels found in the synaptic nerve terminals of the spinal dorsal horn region [4,5]. Ca_V_ channels are divided into three major families named Ca_V_1, Ca_V_2, and Ca_V_3 that have distinct biophysical properties, tissue distributions, and physiological roles [6]. The Ca_V_1 family comprise four different L-type channels (Ca_V_1.1, Ca_V_1.2, Ca_V_1.3, and Ca_V_1.4), the Ca_V_2 family comprise one P/Q-type (Ca_V_2.1), one N-type (Ca_V_2.2), and one R-type (Ca_V_2.3) channel, and the Ca_V_3 family comprise three T-type (Ca_V_3.1, Ca_V_3.2, and Ca_V_3.3) channels [7,8,9].

The Ca_V_2.2 channels play a crucial role in transmitting nociceptive pain signals from the peripheral nervous system (PNS) to the central nervous system (CNS) [10] and are considered as promising analgesic targets. The ability of certain ω-conotoxins to selectively block Ca_V_2.2 channels led to their development as a new class of intrathecal analgesics for severe pain. GVIA, isolated from *Conus geographus*, was reported to be the first ω-conotoxin irreversibly blocking Ca_V_ channel [11]. In 2004, Ziconotide, a synthetic version of ω-conotoxin MVIIA found in *Conus magus*, gained approval from US Food and Drug Administration (FDA) to treat chronic refractory pain [12,13], albeit with dose limiting side effects including dizziness, nystagmus, sedation, blurred vision, and orthostatic hypotension [14,15,16]. Poor oral bioavailability and CNS access combined with a short plasma half-life [17] restrict the use of MVIIA to intrathecal administration with incumbent complications of headaches, pump-associated infections, and bleeding-associated diathesis [18,19]. Other highly potent ω-conotoxins, e.g., CVID and CVIF isolated form *Conus catus*, showed potential as an analgesic with a better safety profile in preclinical studies, with CVID showing uncertain therapeutic advantage in limited clinical studies [20,21,22,23].

Acute postsurgical pain (PSP) and chemotherapy-induced peripheral neuropathy (CIPN) are two major health issues worldwide, related to peripheral pain. Despite receiving acute pain medications, up to 71% of patients report moderate to extreme pain after surgery [24,25,26]. Currently available treatment options to manage PSP include opioid analgesics, nonsteroidal anti-inflammatory drugs, local anesthetics, paracetamol, and physiotherapy. These analgesics have abuse potential, side effects, and even lack of efficacy. On the other hand, CIPN is one of the major side effects of anticancer drugs, with a very high prevalence of 70–100% for platinum-based antineoplastic agents [27]. At present, there is no safe and fully effective treatment for CIPN, with partial relief from pain provided by anticonvulsants, e.g., carbamazepine, gabapentin, and antidepressants, e.g., amitriptyline and duloxetine [28]. Due to increasingly effective anticancer treatments, the number of cancer survivors that suffer from CIPN are increasing every year [29]. To improve the quality of their lives, it is crucial to develop effective therapeutic strategies to treat CIPN.

The central analgesic effects of intrathecal ω-conotoxins have been characterised in a range of rodent pain models [20,30,31,32]. Although previous studies suggest that calcium channels contribute to peripheral neural mechanisms of hyperalgesia [33], the peripheral analgesic effects of ω-conotoxins remain to be fully characterised. In this study, we investigated the analgesic effects of subcutaneously administered ω-conotoxins GVIA, MVIIA, and CVIF in rodent models of postsurgical pain (PSP) and cisplatin- and oxaliplatin-induced peripheral neuropathy (CIPN).

## 2. Results

### 2.1. ω-Conotoxins Inhibit Endogenous Ca_V_2.2 Channel In Vitro and Lack Side Effects In Vivo

**Figure 1:**
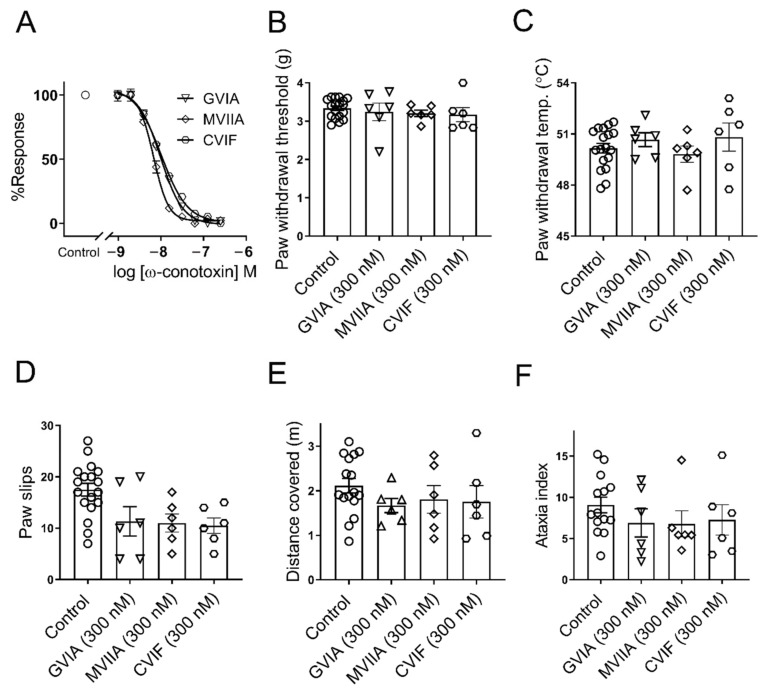
Potency of Ca_V_2.2 channel inhibition and side effects of intraplantar administration of ω-conotoxins. (**A**) Representative concentration response curves for inhibition of KCl evoked Ca_V_2.2 response in SH-SY5Y cells. Potency of GVIA (IC_50_ 11.2 ± 3.3 nM), MVIIA (IC_50_ 6.8 ± 2.1 nM), and CVIF (IC_50_ 10.0 ± 3.1 nM) determined from three independent experiments each conducted in triplicate. (**B**,**C**) Intraplantar injection of 6 pmol/paw (300 nM; 20 µL) GVIA, MVIIA, and CVIF (*n* = 6 per group) in naïve C57BL6/J mice did not significantly change mechanical and thermal PWTs compared to healthy control group (*n* = 18) (*p* > 0.05). (**D**–**F**) Intraplantar injection of 6 pmol/paw (300 nM; 20 µL) GVIA, MVIIA, and CVIF (*n* = 6 per group) in naïve C57BL6/J mice did not significantly change foot slips, distance covered, and ataxia index in the parallel rod floor test compared with healthy control mice (*n* = 18) (*p* > 0.05). All data are presented as mean ± SEM. Statistical significance was determined using one-way ANOVA with Dunnett’s post-test.

We determined the potency of GVIA, MVIIA, and CVIF to inhibit Ca_V_2.2 channel response by measuring the fluorescence signals from endogenous Ca_V_2.2 channels, expressed in the SH-SY5Y cells, using FLIPR^TETRA^. The IC_50_s for Ca_V_2.2 channel inhibition by GVIA, MVIIA, and CVIF were found to be 11.2 ± 3.3, 6.8 ± 2.1, and 10.0 ± 3.1 nM, respectively (Figure 1A).

To investigate the effects of local administration of ω-conotoxins on basal mechanical and thermal pain thresholds, we administered 6 pmol/paw (300 nM; 20 µL) GVIA, MVIIA, and CVIF via intraplantar (i.pl.) injection. The administration of 300 nM GVIA, MVIIA, and CVIF (i.pl.) into naïve mice did not alter mechanical (300 nM GVIA, 3.2 ± 0.2 g; 300 nM MVIIA, 3.2 ± 0.1 g; 300 nM CVIF, 3.1 ± 0.2 g; vehicle control, 3.3 ± 0.1 g; *p* > 0.05; Figure 1B), or thermal (300 nM GVIA, 50.7 ± 0.5 °C; 300 nM MVIIA, 49.8 ± 0.5 °C; 300 nM CVIF, 50.8 ± 0.8 °C; vehicle control, 50.1 ± 0.3 °C; *p* > 0.05; Figure 1C) PWTs compared to vehicle control mice. The data summarising the effects of ω-conotoxins on mechanical PWTs of healthy mice are shown in Supplementary Tables S1 and S2.

The effects of intraplantar administration of ω-conotoxins on locomotor activity were tested by measuring the number of paw slips, and the distance covered over 2 min at 1 h after the administration of a 6 pmol/paw (300 nM; 20 µL) dose of GVIA, MVIIA, and CVIF (i.pl.) (Figure 1D–E). The number of paw slips of GVIA, MVIIA, and CVIF treated mice were not significantly different compared to vehicle treated mice (300 nM GVIA, 11.3 ± 2.8; 300 nM MVIIA, 11.0 ± 1.8 g; 300 nM CVIF, 10.5 ± 1.5 g; vehicle control, 17.0 ± 1.5 g; *p* > 0.05; Figure 1D). The distances covered by GVIA, MVIIA, and CVIF treated mice were also not significantly different compared to vehicle treated mice (300 nM GVIA, 1.7 ± 0.2 m; 300 nM MVIIA, 1.8 ± 0.3 m; 300 nM CVIF, 1.7 ± 0.4 m; vehicle control, 2.1 ± 0.2 m; *p* > 0.05; Figure 1E). No significant change in ataxia index (paw slips/distance covered) was observed between GVIA, MVIIA, and CVIF treated mice compared to vehicle treated mice (300 nM GVIA, 6.9 ± 1.7 m; 300 nM MVIIA, 6.7 ± 1.6 m; 300 nM CVIF, 7.3 ± 1.8 m; vehicle control, 9.1 ± 0.1 m; *p* > 0.05; Figure 1F). The data of the locomotor side effects of ω-conotoxins are summarized in Supplementary Tables S3–S5.

### 2.2. Local Effects of ω-Conotoxins on Surgery-Induced Mechanical and Thermal Allodynia

**Figure 2:**
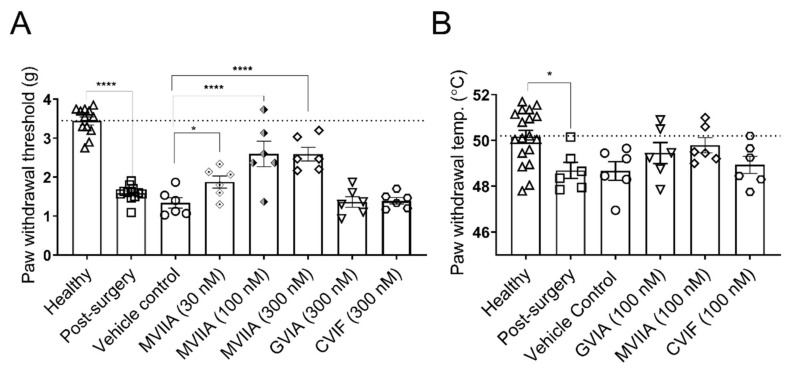
Analgesic effects of ω-conotoxins on surgery-induced mechanical and thermal allodynia. (**A**) Surgery-induced mechanical allodynia developed 24 h after surgery. Paw withdrawal thresholds were significantly lowered in the surgery group compared with naïve mice (*p* < 0.0001; *n* = 12 per group). Compared with vehicle control, the PWTs of post-surgery mice increased significantly after intraplantar injection of 0.6, 2.0, and 6.0 pmol/paw (30, 100, and 300 nM MVIIA; 20 µL) MVIIA (*p* < 0.05; *p* < 0.0001; *p* < 0.0001, respectively; *n* = 6 per group), but remained unchanged after intraplantar injection of 6.0 pmol/paw (300 nM; 20 µL) GVIA and CVIF (*p* > 0.05; *n* = 6 per group). (**B**) The thermal thresholds of mice 24 h after surgery were significantly lower, compared with naïve mice (*p* < 0.05; *n* = 18). However, the intraplantar injection of 2.0 pmol/paw (100 nM; 20 µL) GVIA, MVIIA, and CVIF did not reverse thermal allodynia in mice 24 h after surgery compared to vehicle control group (*p* > 0.05; *n* = 6 per group). All data are presented as mean ± SEM. Statistical significance was determined using one-way ANOVA with Dunnett’s post-test; * *p* < 0.05; **** *p* < 0.0001 compared with vehicle control group (except where indicated otherwise).

Surgery of the right hind paw elicited mechanical allodynia in mice, with PWTs to mechanical stimulation of mice in the surgery group being significantly lower compared to naïve mice at 24 h after surgery (naïve, 3.4 ± 0.1 g; post-surgery, 1.5 ± 0.1 g; *p* < 0.0001; Figure 2A). All animals maintained body weight and a healthy appearance. The animals were monitored during the whole time and no sign of adverse effects of the surgery was observed.

Intraplantar administration of 2 pmol/paw (100 nM; 20 µL), and 6 pmol/paw (300 nM; 20 µL) MVIIA significantly increased mechanical PWTs compared to vehicle control (100 nM MVIIA, 2.6 ± 0.3 g; 300 nM MVIIA, 2.6 ± 0.2 g; vehicle control, 1.3 ± 0.2 g; *p* < 0.0001; Figure 2A). Impressively, doses as low as 0.6 pmol/paw (30 nM; 20 µL) (i.pl.) MVIIA significantly increased mechanical PWTs compared to vehicle control (30 nM MVIIA, 1.9 ± 0.2 g; vehicle control, 1.3 ± 0.2 g; *p* < 0.05; Figure 2A). In contrast, 6 pmol/paw (300 nM; 20 µL) GVIA and CVIF (i.pl.) did not produce any significant antiallodynic effect compared to vehicle control (300 nM GVIA, 1.4 ± 0.1 g; 300 nM CVIF, 1.4 ± 0.1 g; vehicle control, 1.3 ± 0.2 g; *p* > 0.05; Figure 2A).

Surgery also caused small but significant thermal allodynia in mice, with the PWTs to thermal stimulation of mice with incised paws being significantly lower compared to naïve mice, 24 h after surgery (naïve, 50.2 ± 0.3 °C; post-surgery, 48.7 °C ± 0.4 g; *p* < 0.05; Figure 2B). However, none of the ω-conotoxins effectively reduced thermal allodynia when administered via intraplantar injection in a dose of 2 pmol/paw (100 nM; 20 µL) compared to control mice (100 nM GVIA, 49.5 ± 0.5 °C; 100 nM MVIIA, 49.8 ± 0.3 °C; 100 nM CVIF, 48.9 ± 0.4 °C; vehicle control, 48.7 ± 0.4 °C; *p* > 0.05; Figure 2B). The data of surgery-induced mechanical and thermal allodynia are summarized in Supplementary Tables S6 and S7.

### 2.3. Local Effects of ω-Conotoxins on Chemotherapy-Induced Mechanical Allodynia

#### 2.3.1. Effect of ω-Conotoxins in Oxaliplatin-Induced Mechanical Allodynia

**Figure 3:**
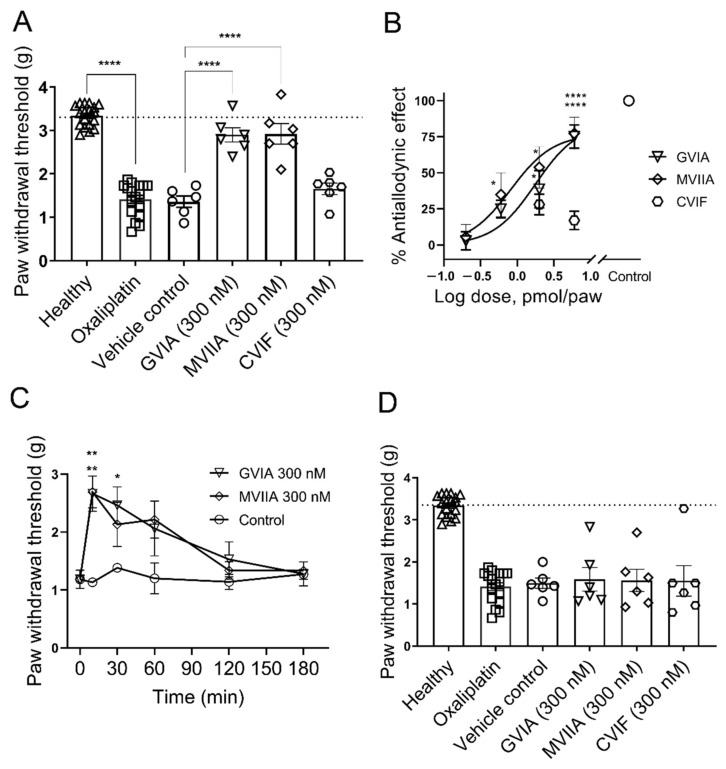
Analgesic effects of ω-conotoxins in oxaliplatin-induced mechanical allodynia. (**A**) Mechanical allodynia developed 24 h after intraplantar administration of 40 µg/paw oxaliplatin. Paw withdrawal thresholds were significantly reduced in oxaliplatin injected group compared to naïve mice (*p* < 0.0001; *n* = 18 per group). Administration of 6 pmol/paw (300 nM; 20 µL) GVIA and MVIIA significantly increased the PWTs of oxaliplatin-treated mice compared to vehicle control group (*p* < 0.05, *p* < 0.0001 *n* = 6 per group). However, 6 pmol/paw (300 nM; 20 µL) CVIF did not significantly change PWTs compared to vehicle control mice (*p* > 0.05; *n* = 6 per group). (**B**) Dose response curves of the antiallodynic effect of GVIA and MVIIA on oxaliplatin-induced mechanical allodynia. The ED_50_ of GVIA and MVIIA was found to be 1.8 pmol/paw and 0.8 pmol/paw, respectively. Data indicated by the hexagons are from two doses (2 and 6 pmol/paw) (100 and 300 nM; 20 µL) of CVIF (*n* = 6 per data point). (**C**) Time course of action of GVIA and MVIIA in the OIPN model. Paw withdrawal thresholds were measured after intraplantar injection of 6 pmol/paw (300 nM; 20 µL) GVIA and MVIIA. 300 nM GVIA significantly increased PWTs at 10 (*p* < 0.01; *n* = 6 per group) and 30 min (*p* < 0.05; *n* = 6 per group) after injection while 300 nM MVIIA significantly increased PWTs only at 10 min (*p* < 0.01; *n* = 6 per group) after injection, compared with vehicle control group. The increase in mechanical PWTs produced by GVIA and MVIIA gradually returned to pre-injection levels at 120–180 min after treatment. (**D**) Contralateral administration of 6 pmol/paw (300 nM; 20 µL) GVIA, MVIIA, and CVIF, 24 h after intraplantar administration of oxaliplatin, did not significantly change mechanical PWTs compared to vehicle treated control group (*p* > 0.05; *n* = 6 per group). All data are presented as mean ± SEM. Statistical significance was determined using one-way ANOVA with Dunnett’s post-test, or two-way ANOVA with Sidak’s post-test as appropriate; * *p* < 0.05; ** *p* < 0.01, **** *p* < 0.0001 compared with vehicle control (except where indicated otherwise).

The intraplantar administration of a single dose (40 µg/paw) of oxaliplatin into the right hind paw elicited strong mechanical allodynia 24 h after injection (Figure 3A) (control, 3.3 ± 0.1 g; oxaliplatin, 1.4 ± 0.1 g; *p* < 0.0001; Figure 3A). Oxaliplatin injected mice maintained healthy appearance and body weight with no sign of licking, shaking, or lameness of the injected paw.

We assessed the antiallodynic effects of intraplantar injection of ω-conotoxin GVIA, MVIIA, and CVIF on oxaliplatin-induced mechanical allodynia (Figure 3A–D). Administration of 6 pmol/paw (300 nM; 20 µL) GVIA and MVIIA (i.pl.) led to a reversal of mechanical allodynia, which was significantly higher than vehicle control mice (300 nM GVIA, 2.9 ± 0.2 g; 300 nM MVIIA, 2.9 ± 0.2 g; vehicle control, 1.4 ± 0.1 g; *p* < 0.0001; Figure 3A) but not different from naïve mice (naïve, 3.3 ± 0.1 g; *p* > 0.05; Figure 3A). On the contrary, 6 pmol/paw (300 nM; 20 µL) CVIF (i.pl.) did not elicit significant antiallodynic effect compared to vehicle control (300 nM CVIF, 1.7 ± 0.1 g; vehicle control, 1.4 ± 0.1 g; *p* > 0.05; Figure 3A).

To investigate the effects at lower doses, we tested 2.0, 0.6, and 0.2 pmol/paw (100, 30, and 10 nM; 20 µL) GVIA and MVIIA (i.pl.). Administration of 100 nM GVIA and MVIIA (i.pl.) significantly increased mechanical PWTs compared to vehicle control (100 nM GVIA, 2.1 ± 0.3 g; 100 nM MVIIA, 2.2 ± 0.4 g; vehicle control, 1.4 ± 0.1 g; *p* < 0.05). Although 30 nM MVIIA (i.pl.) significantly increased mechanical PWTs compared to vehicle control (30 nM MVIIA, 2.0 ± 0.3 g; vehicle control, 1.4 ± 0.1 g; *p* < 0.05), 30 nM GVIA (i.pl.) did not produce any significant antiallodynic effect compared to vehicle control (30 nM GVIA, 1.8 ± 0.1 g; vehicle control, 1.4 ± 0.1 g; *p* > 0.05). Neither 10 nM GVIA (i.pl.) nor 10 nM MVIIA (i.pl.) produced any significant antiallodynic effect compared to vehicle control (10 nM GVIA, 1.4 ± 0.1 g; 10 nM MVIIA, 1.4 ± 0.2 g; vehicle control, 1.4 ± 0.1 g; *p* > 0.05). These data are summarized in Supplementary Table S8. Both GVIA and MVIIA elicited dose-dependent reversal of mechanical allodynia in the model of OIPN. From the dose response curves, the ED_50_ of the antiallodynic effect of GVIA (1.8 pmol/paw, 95% CI 0.6–3.7 pmol/paw) and MVIIA (0.8 pmol/paw, 95% CI 0.3–2.5 pmol/paw) were not significantly different. However, these ED_50_ values were determined from four-point dose-response curves and assume these ω-conotoxins do not have an analgesic component in addition to their antinociceptive effect.

The time course of the effect of the locally administered 6 pmol/paw (300 nM; 20 µL) GVIA and MVIIA (i.pl.) were examined 24 h after administration of oxaliplatin (40 µg/paw). GVIA elicited an increase in mechanical PWTs that was significantly higher compared to vehicle at 10 min (300 nM GVIA, 2.7 ± 0.3 g; vehicle control, 1.1 ± 0.1 g; *p* < 0.01; Figure 3C), and 30 min (300 nM GVIA, 2.5 ± 0.3 g; vehicle control, 1.4 ± 0.1 g; *p* < 0.05; Figure 3C) after injection. However, 300 nM MVIIA significantly increased mechanical PWTs only at 10 min (300 nM MVIIA, 2.7 ± 0.3 g; vehicle control, 1.1 ± 0.1 g; *p* < 0.01; Figure 3C) after injection. Both effects were reversible at 120 min after injection.

We also investigated the systemic effects of intraplantar administration of ω-conotoxins on OIPN by contralateral administration of GVIA, MVIIA, and CVIF, 24 h after the administration of oxaliplatin (40µg/paw; i.pl.) into the right hind paw. Contralateral administration of 6 pmol/paw (300 nM; 20 µL) GVIA, MVIIA, and CVIF (i.pl.) did not significantly alter the mechanical PWTs compared to vehicle (300 nM GVIA, 1.6 ± 0.2 g; 300 nM MVIIA, 1.5 ± 0.2 g; 300 nM CVIF, 1.6 ± 0.4 g; vehicle control, 1.5 ± 0.1 g; *p* > 0.05; Figure 3D). The data of the contralateral effects of ω-conotoxins in mechanical PWTs are summarized in Supplementary Table S9.

#### 2.3.2. Effect of ω-Conotoxins in Cisplatin-Induced Mechanical Allodynia

**Figure 4:**
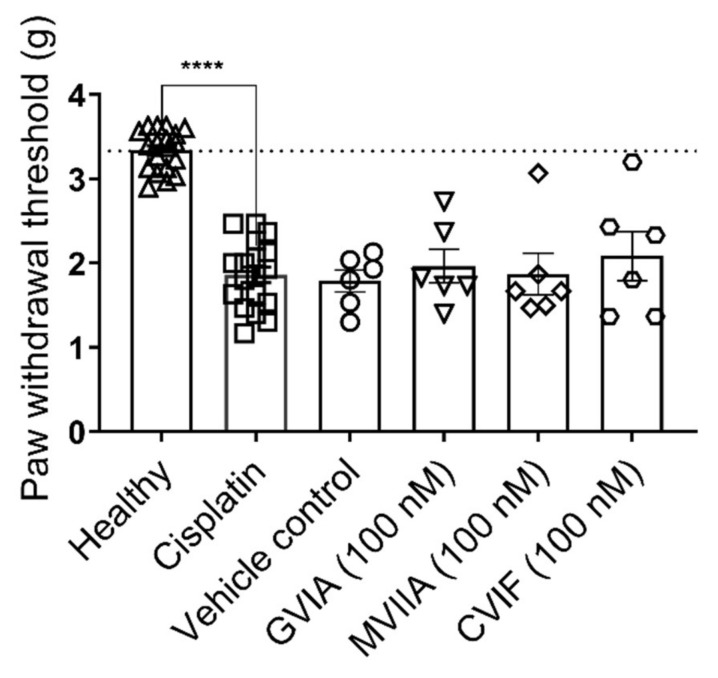
Analgesic effects of ω-conotoxins in cisplatin-induced mechanical allodynia. Cisplatin-induced mechanical allodynia developed 24 h after intraplantar administration of 40 µg/paw cisplatin. Paw withdrawal thresholds were significantly reduced in cisplatin injected group compared to naïve mice (*p* < 0.0001; one-way ANOVA; *n* = 18 per group). Intraplantar injection of 2 pmol/paw (100 nM; 20 µL) GVIA, MVIIA, and CVIF did not significantly change the PWTs in cisplatin-treated mice compared to vehicle control mice (*p* > 0.05; *n* = 6 per group). All data are presented as mean ± SEM. Statistical significance was determined using one-way ANOVA with Dunnett’s post-test, **** *p* < 0.0001 compared with vehicle control (except where indicated otherwise).

Intraplantar administration of 40 µg/paw cisplatin into the right hind paw elicited strong mechanical allodynia in mice 24 h after the injection (Figure 4). The mechanical paw withdrawal thresholds of mice injected with cisplatin were significantly lower compared to naïve mice when tested 24 h after injection (naïve, 3.3 ± 0.1 g; 40 µg/paw cisplatin, 1.9 ± 0.1 g; *p* < 0.0001; Figure 4). Cisplatin injected mice did not lose weight and maintained a healthy appearance. No sign of adverse effects including, licking, shaking, or lameness of the injected paws was observed.

We assessed the analgesic effects of intraplantar injection of 2 pmol/paw (100 nM, 20 µL) GVIA, MVIIA, and CVIF against cisplatin-induced mechanical allodynia (Figure 4). Administration of 100 nM MVIIA, GVIA, and CVIF did not produce any significant antiallodynic effect compared to vehicle control (100 nM GVIA, 2.0 ± 0.2 g; 100 nM MVIIA, 1.9 ± 0.2 g; 100 nM CVIF, 2.0 ± 0.3 g; vehicle control, 1.8 ± 0.2 g; *p* > 0.05; Figure 4).

## 3. Discussion

This study has demonstrated that the intraplantar administration of ω-conotoxins effectively alleviate acute peripheral neuropathy in different mouse models of acute and neuropathic pain. We found that the administration of up to 0.6 pmol/paw MVIIA (i.pl.) elicited an increase of mechanical PWTs in mice, which had undergone surgery on the plantar foot through the skin and fascia. Small but significant heat allodynia was elicited at 24 h after the incision which was consistent with previous findings [34]. However, none of the ω-conotoxins changed thermal PWTs compared to vehicle control after surgery. This lack of activity was observed in previous studies, where intrathecal administrations of MVIIA and CVID were found to be ineffective to reduce thermal allodynia in rodent model of neuropathic pain [21]. These findings were not surprising, considering the differences in the physiological properties of the nociceptors contributing to heat or mechanical hyperalgesia after incision, [35]. Heat hyperalgesia depends mainly on the activation of spinal N-methyl-D-aspartate (NMDA) receptors, transfer of protein kinase C, and production of cGMP and nitric oxide. In contrast, mechanical hyperalgesia is elicited by co-activation of metabotropic glutamate receptors and spinal α-amino-3-hydroxy-5-methy-4-isoxazole propionate (AMPA), activation of phospholipase A2 (PLA2), and production of cyclooxygenase products [36,37,38,39,40].

In our study, the intraplantar application of GVIA and MVIIA significantly alleviated mechanical pain in OIPN model but was ineffective in CisIPN model suggesting differential mechanisms leading to development of OIPN and CisIPN. Although both oxaliplatin and cisplatin are platinum based antineoplastic drugs, and both act by DNA alkylation [41,42,43], they differ markedly in their ability to elicit pain syndromes [44]. Oxaliplatin causes both mechanical and cold allodynia, while cisplatin only evokes mechanical allodynia [45,46,47]. The most distinctive feature of oxaliplatin is its fast, non-enzymatic transformation to oxalate and dichloro 1,2-diaminocyclohexyl-platinum complex, which do not contribute to the cold but mechanical allodynia [48]. Moreover, intraplantar administration of oxaliplatin and cisplatin causes the over-expression of different sets of genes that could be involved in development of neuropathic pain following oxaliplatin and cisplatin administration in mouse dorsal root ganglia [44]. However, the exact mechanisms of CisIPN and OIPN are still unknown and differential characteristics of cisplatin-, and oxaliplatin-induced pain indicates different mechanisms might be involved [45]. 

The time course of action of subcutaneously administered ω-conotoxins has previously been shown to differ from intrathecally administered ω-conotoxins in spinal nerve ligation (SNL) model of neuropathic pain [21]. In our study, the peak response after intraplantar administration of GVIA and MVIIA (300 nM) was achieved within 10 min and it returned to pre-injection levels in less than 2 h. However, in the SNL model, the peak response was achieved 2 h after the intrathecal administration of GVIA and MVIIA, and residual effect was present at 24 to 48 h, especially at higher dose (1 µg/kg) [21]. This difference is probably due to the different pharmacokinetics followed by the ω-conotoxins after intraplantar and intrathecal administration. However, the contralateral administration of GVIA, MVIIA, and CVIF (300 nM; i.pl.) did not reverse mechanical allodynia in our OIPN model, suggesting that subcutaneously administered ω-conotoxins lack systemic analgesic effects within 10 min in the OIPN model at the doses administered. Supporting a lack of systemic effects, intraplantar administration of 300 nM GVIA, MVIIA or CVIF did not elicit ataxia or locomotion-related side effects e.g., paw slips, and distance travelled determined at 1 h after administration in mice. Previously, subcutaneous administration of ω-conotoxin CVID, CVIE and CVIF near the scruff of the neck did not cause side effects, although it partially reversed the incapacitance of mouse inflammatory pain [27,45,46,49,50,51].

Although we have not investigated the site of action in this study, considering the high selectivity to Ca_V_2.2 channels, it is likely that the peripheral analgesic activity of ω-conotoxins in PSP and CIPN is due to the inhibition of Ca_V_2.2 channels in peripheral nociceptors. Earlier studies have shown the involvement of peripheral CaV channels in pain signaling. Previously, Sann et al. demonstrated that, in *Xenopus laevis* embryos, Ca_V_2.2 channels are expressed in sensory axons innervating the skin [50] and recent studies have shown that in mice, Ca_V_ channels are present along peripheral nerve axons, and Ca^2+^ currents in peripheral nerves are mediated mainly by Ca_V_2.2 and Ca_V_1.1 channels [51]. Moreover, it has been reported that increased local concentrations of Ca^2+^ are involved in nerve injury-induced hyperalgesia [52,53]. This hypothesis is consistent with the study of Cousins et al. where nerve injury-induced hyperalgesia in mice was attenuated by subcutaneous application of MVIIA, while the selective Ca_V_1.1 blocker nifedipine or the selective Ca_V_2.1 blocker MVIIC were ineffective [33].

In the current study, oxaliplatin-induced mechanical pain was effectively reduced by both GVIA and MVIIA (i.pl.), but surgery-induced mechanical pain was alleviated only by MVIIA (i.pl.). Interestingly, CVIF (i.pl.) was not found effective in any of the peripheral pain models. This contrasting action of ω-conotoxins is particularly intriguing as all the ω-conotoxins blocked Ca_V_2.2 responses from SH-SY5Y cells with similar potency in FLIPR assay (IC_50_ for GVIA, MVIIA, and CVIF inhibition was 6.4 ± 2.2 nM, 9.1 ± 3.1 nM, and 8.6 ± 2.9 nM, respectively). In a previous study, different concentration-dependent inhibition of Ca_V_2.2 response from different ω-conotoxins was reported by Adams et al., where CVID but not MVIIA, inhibited a distinctive N-type calcium channel to stop nerve-evoked transmitter release from preganglionic cholinergic nerves innervating the rat submandibular ganglia [54].

Although the exact reason behind the difference in Ca_V_2.2 channel inhibition by ω-conotoxins is unclear, there are several possibilities. Previously, Mould et al. reported that the presence of auxiliary Ca_V_ subunit, α_2_δ, reduce the affinity of the N-type channels for the ω-conotoxins, varying from 150–680-fold, where the effect was more prominent for CVID compared with MVIIA [55]. Several studies have shown that the α_2_δ subunit is critically involved in neuropathic pain as the expression of α_2_δ_1_ subunit increase significantly in dorsal root ganglia (DRG) in neuropathic pain models, [56,57,58,59]. In addition, the α_2_δ subunit is highly glycosylated [60], and the glycosylated α_2_ domain may cover the binding site of ω-conotoxin near the channel by creating electrostatic shielding [61]. Even though the effect of glycosylation in ω-conotoxin binding has not been thoroughly studied, differential glycosylation might contribute to the differences observed. Another possible reason might be linked to the presence of a different splice variant of Ca_V_2.2 in peripheral nerve axons near skin, given single residues in domain III extracellular loop of Ca_V_2.2 can affect ω-conotoxin access [62]. GVIA and MVIIA block resting, open, and inactivated channels [63], whereas CVIF has a higher affinity for channels in the inactivated state [23]. Thus, state dependence of channel block might influence the effectiveness of ω-conotoxins in different pain models.

The finding that local (subcutaneous) application of ω-conotoxins have analgesic potential in mouse models of PSP and OIPN without locomotor side effects, indicates, this class of analgesic has clinical potential for the treatment of postsurgical pain and OIPN. Peptide based drugs are increasingly gaining acceptance as clinical leads due to their high specificity and potency, although often lower bioavailability, metabolic instability and membrane permeability can limit their use [64]. In our study we found that onset of analgesic action of GVIA and MVIIA on OIPN was rapid (< 10 min) although the duration of action at the dose investigated was also short (60 min). However, to prolong the analgesic activity, controlled drug delivery systems like microspheres, hydrogels, liposomes, or solid-lipid nanoparticles can be implemented [65]. In addition, further studies are required to understand the mechanism behind differential effects of GVIA, MVIIA and CVIF in the skin in different models of pain.

## 4. Materials and Methods

### 4.1. Chemicals

Cisplatin and oxaliplatin were obtained from Sigma Aldrich (Castle Hill, New South Wales, Australia). The ω-conotoxins GVIA, MVIIA, and CVIF were obtained from Alomone Labs (Jerusalem, Israel). All reagents were from Sigma Aldrich unless otherwise stated.

### 4.2. Cell Culture

The human neuroblastoma cell line SH-SY5Y was cultured in 5% CO_2_ at 37 °C in Roswell Park Memorial Institute (RPMI) (Gibco, Life Technologies, Carlsbad, CA, USA) supplemented with 15% (*v*/*v*) fetal bovine serum (FBS) and 1 mM L-glutamine. Dulbecco’s phosphate-buffered saline (DPBS) (Gibco, Life Technologies, Carlsbad, CA, USA), and 0.25% Trypsin-EDTA were used to wash and detach the cells from the flask, respectively.

### 4.3. Animals

All behavioral experiments (CisIPN, OIPN, and PSP models) were performed using adult male C57BL/6J mice (age 8–10 weeks). The mice were housed in groups of 3 or 4 per cage under 12-h light–dark cycles with access to water and standard rodent chow ad libitum. Ethical approvals for in vivo experiments in animals were obtained from the University of Queensland animal ethics committee. The animal ethics approval numbers for postsurgical pain model and cisplatin- and oxaliplatin-induced neuropathy model were PHARM/170/16 and PHARM/PACE/368/16. The experiments were conducted in accordance with the Australian Code of Practice for the Care and Use of Animals for Scientific Purposes, eighth edition (2013) and the International Association for the Study of Pain Guidelines for the Use of Animals in Research. Animals were kept in the experimental room for at least 30 min before behavioral testing was performed. All measurements were performed at room temperature (ambient temperature of 21–23 °C) by a blinded observer kept unaware of the treatment each animal received. Sample sizes of each experiment are detailed in the figure legends of the corresponding figure. Statistical justification for *n* = 6 per group is as follows: 80% power to detect a 30% difference relative to baseline readings with a 25% interindividual variability between animals with a statistical significance criterion of *p* < 0.05.

### 4.4. Fluorescence-Imaging Assays

The increase of intracellular calcium induced by the Ca_V_2.2 channel activation was measured by using calcium-sensitive Calcium 4 no-wash kit (Molecular Devices, Sunnyvale, CA, USA). Fluorescence signals were detected using a fourth generation Fluorescence Imaging Plate Reader, FLIPR^TETRA^ (Molecular Devices, Sunnyvale, CA, USA). Cells were seeded on 384-well black-walled clear flat-bottom imaging plates (Corning, Lowell, MA, USA) at a density of 50,000 cells/well, 24 h before the assay. The Calcium 4 dye was diluted in physiological salt solution (PSS; 140 mM NaCl, 11.5 mM glucose, 5.9 mM KCl, 1.4 mM MgCl2, 1.2 mM NaH_2_PO_4_, 5 mM NaHCO_3_, 1.8 mM CaCl_2_, 10 mM HEPES, pH 7.4) with 0.1% bovine serum albumin (BSA), with 10 µM nifedipine added to block the native L-type Ca_V_ response in SH-SY5Y cells. The media was removed, and the cells were loaded with 20 µL dye per well and incubated for 30 min at 37 °C in a 5% humidified CO_2_ incubator. Excitation and emission wavelength of FLIPR^TETRA^ were set at 470–495 and 515–575 nM, respectively, and camera gain and intensity adjusted for each plate to yield a minimum of 1500–2000 arbitrary fluorescence units (AFU) baseline fluorescence. A two-addition protocol was used where 5 baseline fluorescence readings were taken prior to first addition of 10 µL ω-conotoxins (1–500 nM) followed by fluorescent readings for 10 min. In the second addition, 10 µL stimulation buffer containing 90 mM KCl and 5 mM CaCl_2_ was added, followed by a further 10 min of fluorescent readings. Although the addition of 90 mM KCl and 5 mM CaCl_2_ leads to changes in osmolarity, it does not significantly affect Ca^2+^ transients, as illustrated by the lack of effect of addition of 90 mM NaCl and 5 mM CaCl_2_, which affects osmolarity to the same extent (Supplementary Figure S1). To normalize the data, the response from positive control (90 mM KCl and 5 mM CaCl_2_) was defined as 100% and the response from negative control (PSS buffer) was defined as 0%.

### 4.5. Cisplatin- and Oxaliplatin-Induced Neuropathy

Previously established models of cisplatin- and oxaliplatin-induced neuropathy [66] were used to assess the effect of GIVA, MVIIA, and CVID on mechanical allodynia. Briefly, a single dose of 40 µL cisplatin or oxaliplatin was administered by shallow intraplantar injection (i.pl.) to the right hind paw under light isoflurane anaesthesia to induce peripheral neuropathy in mice, as described previously [66].

### 4.6. Postsurgical Pain

Surgery of mice was performed following a previously described method [34]. Mice were anaesthetized by inhalation with 3% isoflurane for induction, followed by 1–1.5% isoflurane for maintained anaesthesia via a nose cone. Seventy percent ethanol was used to disinfect the plantar (glabrous) part of the right hind paw before a longitudinal incision of 7 mm was made on the plantar surface of the hind paw through the skin and fascia using a number 11 surgical scalpel. The incision started from about 3 mm proximal point of the heel and extended toward the toes. Keeping the origin and insertion intact, the plantaris muscle was incised longitudinally and elevated with sterile forceps to mimic muscle retraction. Gentle pressure with sterile gauze was applied to the wound to stop bleeding. The incision was closed with two sterile sutures after hemostasis, using the simple interrupted suture technique, and Betadine (5% povidone–iodine solution) was spread over the closed wound. After stopping inhalation anaesthesia, the mice were observed for abnormal behaviours, including signs of spontaneous pain and paw guarding for 10 min. Finally, mice were moved back to their cages for recovery prior to behavioural assessments.

### 4.7. Mechanical Paw Withdrawal Threshold Measurements

Mechanical paw withdrawal thresholds (PWT) were assessed using electronic von Frey apparatus (MouseMet Electronic von Frey, Topcat Metrology Ltd., Little Downham, United Kingdom) as described previously [62,67,68,69]. Briefly, animals were habituated in individual mouse runs for at least 30 min before the measurements. Using a soft-tipped von Frey filament, the pressure against the foot pad of the right hind paw was slowly increased through rotation of the device handle with the force increasing at a rate of 1 g/s, and the force (g) causing paw withdrawal (lift, shake, lick) was displayed by the apparatus. For the PSP model, the von Frey probe tip was placed adjacent to the wound, against the glabrous plantar surface. One biological replicate was determined by averaging three repeated measurements at 5 min intervals for each mouse. Three repeats were consistent and there was no sign of sensitization or desensitization. Mechanical allodynia was assessed 24 h after injection of cisplatin and oxaliplatin, and 24 h after surgery. To normalize the data for the dose response curves of ω-conotoxins in OIPN, the response from vehicle control was defined as 0% and the response from healthy control was defined as 100%. No constraints were added to define the bottom of the curves, but the top of the curve was set to “must be less than 100” considering ω-conotoxins typically do not show analgesic effects in addition to their antinociceptive actions.

### 4.8. Thermal Paw Withdrawal Threshold Measurements

Thermal PWT was assessed using the MouseMet Thermal apparatus (Topcat Metrology Ltd., Little Downham, United Kingdom) as described previously [70]. Briefly, at least 30 min prior testing, mice were placed in individual mouse runs to acclimatize to the room conditions. The plantar side of the incised right hind paw was touched by the tip of the probe which was preheated to 37 °C. The heat was automatically increased at a rate of 2.5 °C/s, and the temperature limit was set to 55 °C. One biological replicate was determined by averaging three repeated measurements at 5 min intervals for each mouse, and heat thresholds were measured 24 h after surgery. Three repeats were consistent and there was no sign of sensitization or desensitization.

### 4.9. Locomotor Performance Assessment

Motor coordination and locomotor effects of intraplantar administration of ω-conotoxins were measured using a Parallel Rod Floor apparatus (Stoelting Co, Wood Dale, IL, USA) by assessing foot slips and distance covered as described previously [68]. The number of foot slips and the distance travelled (m) were assessed for 2 min at 1 h after the intraplantar injection of ω-conotoxins. The ataxia index was calculated by dividing the number of foot slips by the distance travelled (m), and the ANY-Maze software (Stoelting Co) was used to records the foot slips and the distance travelled.

### 4.10. Treatments

To avoid spontaneous hydrolysis caused by Cl^−^ in physiological solutions, cisplatin and oxaliplatin were prepared on the day of the experiment in 5% glucose/H_2_O solution. On the day of the experiment, ω-conotoxin GVIA, MVIIA, and CVIF were prepared in phosphate-buffered saline with 0.1% bovine serum albumin (BSA) to keep them free from adsorption to plastics. Forty microliters of 1 µg/µL cisplatin and oxaliplatin were administered by intraplantar injection into the right hind paw through a sterile 30 G needle, as described previously [68]. To investigate local effects, the ω-conotoxins were administered ipsilateral in a volume of 20 µL/paw using a sterile 30 G needle, 24 h after intraplantar injection of cisplatin and oxaliplatin, and 24 h after surgery. To investigate systemic effects, GVIA, MVIIA, and CVIF were administered by intraplantar injection, contralateral to oxaliplatin administration. Mechanical and thermal allodynia were measured 10 min following the intraplantar administrations of ω-conotoxins. Locomotor side effects are at least partially controlled by CNS [70], and previous studies have already proved that intrathecally administered ω-conotoxins can show analgesic effects for more than 1 h [35], therefore, the locomotor side effects were measured 1 h after the administration of ω-conotoxins.

### 4.11. Data and Statistical Analysis

Data analysis was performed using GraphPad Prism v8.0 (GraphPad Software Inc., San Diego, CA, USA). Sigmoidal curves for the calculation of half-maximal inhibitory concentration (IC_50_) values were fitted to individual data points for concentration-responses by a four-parameter logistic Hill equation. The response over baseline from calcium influx fluorescence in FLIPR^TETRA^ was calculated using Screen Works 3.2.0.14. Statistical significance was determined by one-way ANOVA with Dunnett’s post-test, or two-way ANOVA with Sidak’s post-test as appropriate, and *p* < 0.05 was considered significant, unless stated otherwise. Data are presented as the mean ± SEM.

## Supplementary Materials

The following are available online at https://www.mdpi.com/1660-3397/19/2/106/s1, Figure S1: Hypertonic stimulation on SH-SY5Y responses after addition of different depolarizing solutions., Table S1: Effects of ω-conotoxins in mechanical PWTs of healthy mice, Table S2: Effects of ω-conotoxins in thermal PWTs of healthy mice, Table S3: Locomotor side-effects (paw slips) of ω-conotoxins in healthy mice, Table S4: Locomotor side-effects (distance covered) of ω-conotoxins in healthy mice, Table S5: Locomotor side-effects (ataxia index) of ω-conotoxins in healthy mice, Table S6: Effects of ω-conotoxins in surgery-induced mechanical allodynia, Table S7: Effects of ω-conotoxins in surgery-induced thermal allodynia, Table S8: Effects of ω-conotoxins in oxaliplatin-induced mechanical allodynia, Table S9: Contralateral effects of ω-conotoxins in OIPN, Table S10: Effects of ω-conotoxins in cisplatin-induced mechanical allodynia.
