# Supplementary material for: Subcutaneous ω-Conotoxins Alleviate Mechanical Pain in Rodent Models of Acute Peripheral Neuropathy
Source: Mar Drugs. 2021 Feb 11;19(2):106. doi: 10.3390/md19020106 (PMC7917901; doi:10.3390/md19020106)
Supplement: Supplementary file 1 [file marinedrugs-19-00106-s001.pdf]

Table S1: Effects of ω-conotoxins in mechanical PWTs of healthy mice.

Compound	Dose (pmol /paw)	Number of mice (n)	Mechanical PWT (g)		
			Mean ± SEM	95% CI of mean	F (DFn, DFd)
GVIA	6.0	6	3.2 ± 0.2	2.7−3.8	(3, 32) = 0.5
MVIIA	6.0	6	3.2 ± 0.2	3.0−3.4	
CVIF	6.0	6	3.1 ± 0.2	2.7−3.6	
Control	−	18	3.3 ± 0.1	3.2−3.5	

PWT (Paw withdrawal threshold), CI (Confidence interval).

Table S2: Effects of ω-conotoxins in thermal PWTs of healthy mice.

Compound	Dose (pmol /paw)	Number of mice (n)	Thermal PWT (℃)		
			Mean ± SEM	95% CI of mean	F (DFn, DFd)
GVIA	6.0	6	50.7 ± 0.5	49.6−51.7	(3, 32) = 0.8
MVIIA	6.0	6	49.8 ± 0.5	48.6−51.0	
CVIF	6.0	6	50.8 ± 0.8	48.7−52.9	
Control	−	18	50.1 ± 0.3	49.6−50.8	

PWT (Paw withdrawal threshold), CI (Confidence interval).

Table S3: Locomotor side-effects (paw slips) of ω-conotoxins in healthy mice.

Compound	Dose (pmol /paw)	Number of mice (n)	Paw-slips		
			Mean ± SEM	95% CI of mean	F (DFn, DFd)
GVIA	6.0	6	11.3 ± 2.8	4.0− 18.7	(3, 32) = 3.2
MVIIA	6.0	6	11.0 ± 1.8	6.5− 15.5	
CVIF	6.0	6	10.5 ± 1.5	6.6− 14.4	
Control	−	18	17.0 ± 1.5	13.7−20.3	

PWT (Paw withdrawal threshold), CI (Confidence interval).

Table S4: Locomotor side-effects (distance covered) of ω-conotoxins in healthy mice.

Compound	Dose (pmol /paw)	Number of mice (n)	Distance covered (m)		
			Mean ± SEM	95% CI of mean	F (DFn, DFd)
GVIA	6.0	6	1.7 ± 0.2	1.3− 2.1	(3, 32) = 0.8
MVIIA	6.0	6	1.8 ± 0.3	1.0− 2.6	
CVIF	6.0	6	1.7 ± 0.4	0.8− 2.6	
Control	−	18	2.1 ± 0.2	1.8− 2.5	

PWT (Paw withdrawal threshold), CI (Confidence interval).

Table S5: Locomotor side-effects (ataxia index) of ω-conotoxins in healthy mice.

Compound	Dose (pmol /paw)	Number of mice (n)	Ataxia index		
			Mean ± SEM	95% CI of mean	F (DFn, DFd)
GVIA	6.0	6	6.9 ± 1.7	2.5−11.3	(3, 32) = 1.0
MVIIA	6.0	6	6.7 ± 1.6	2.7− 10.9	
CVIF	6.0	6	7.3 ± 1.8	2.5− 12.0	
Control	−	18	9.0 ± 0.9	7.0− 11.1	

PWT (Paw withdrawal threshold), CI (Confidence interval).

Table S6: Effects of ω-conotoxins in surgery-induced mechanical allodynia

Compound	Dose (pmol / paw)	Number of mice (n)	Mechanical PWT (g)		
			Mean ± SEM	95% CI of mean	F (DFn, DFd)
GVIA	6.0	6	1.4 ± 0.1	0.9−1.9	F (5, 30) = 12.4
MVIIA	6.0	6	2.6 ± 0.2****	2.2−3.2	
	2.0	6	2.6 ± 0.3****	1.4−3.7	
	0.6	6	1.9 ± 0.2*	1.3−2.3	
CVIF	6.0	6	1.4 ± 0.1	1.2−1.7	
Control	−	6	1.3 ± 0.2	0.9−1.4	

PWT (Paw withdrawal threshold), CI (Confidence interval). Statistical significance was determined using one-way ANOVA with Dunnett's post-test; *, $P < 0.05$; ****, $P < 0.0001$ compared with vehicle control.

Table S7: Effects of ω-conotoxins in surgery-induced thermal allodynia

Compound	Dose (pm/ paw)	Number of mice (n)	Thermal PWT (℃)		
			Mean ± SEM	95% CI of mean	F (DFn, DFd)
GVIA	2.0	6	49.5 ± 0.5	48.2−50.6	(3, 20) = 1.632
MVIIA	2.0	6	49.8 ± 0.3	48.9−50.6	
CVIF	2.0	6	48.9 ± 0.4	48.0−49.9	
Control	−	6	48.7 ± 0.4	47.6−49.7	

PWT (Paw withdrawal threshold), CI (Confidence interval).

Table S8: Effects of ω-conotoxins in oxaliplatin-induced mechanical allodynia

Compound	Dose (pmol/paw)	Number of mice (n)	Mechanical PWT (g)		
			Mean ± SEM	95% CI of mean	F (DFn, DFd)
GVIA	6.0	6	2.9 ± 0.2****	2.5−3.3	(10, 55) = 12.5
	2.0	6	2.1 ± 0.3*	1.4−2.8	
	0.6	6	1.8 ± 0.1	1.5−2.2	
	0.2	6	1.4 ± 0.1	1.0−1.7	
MVIIA	6.0	6	2.9 ± 0.2****	2.3−3.5	
	2.0	6	2.2 ± 0.4*	1.3−3.0	
	0.6	6	2.0 ± 0.3*	1.2−2.8	
	0.2	6	1.4 ± 0.2	1.0−1.9	
CVIF	6.0	6	1.7 ± 0.1	1.3−2.0	
	2.0	6	1.9 ± 0.2	1.5−2.3	
Control	-	6	1.4 ± 0.1	1.2−1.6	

PWT (Paw withdrawal threshold) described as the mean ± SEM. Statistical significance was determined using one-way ANOVA with Dunnett's post-test; *, $P < 0.05$; ****, $P < 0.0001$ compared with vehicle control.

Table S9: Contralateral effects of ω-conotoxins in OIPN.

Compound	Dose (pmol/paw)	Number of mice (n)	Mechanical PWT (g)		
			Mean ± SEM	95% CI of mean	F (DFn, DFd)
GVIA	6.0	6	1.6 ± 0.2	1.0−2.1	F (3, 20) = 0.1
MVIIA	6.0	6	1.5 ± 0.2	1.0−2.0	
CVIF	6.0	6	1.6 ± 0.4	0.6−2.5	
Control	−	6	1.4 ± 0.1	1.2−1.6	

PWT (Paw withdrawal threshold), CI (Confidence interval).

Table S10: Effects of ω-conotoxins in cisplatin-induced mechanical allodynia

Compound	Dose (pmol/paw)	Number of mice (n)	Mechanical PWT (g)		
			Mean ± SEM	95% CI of mean	F (DFn, DFd)
GVIA	2.0	6	1.6 ± 0.2	1.5−2.5	(3, 20) = 0.2
MVIIA	2.0	6	1.5 ± 0.2	1.2−2.5	
CVIF	2.0	6	1.6 ± 0.4	1.3−2.8	
Control	-	6	1.5 ± 0.1	1.6−2.0	

PWT (Paw withdrawal threshold) described as the mean ± SEM, CI (Confidence interval)
